# Selective deletion of the receptor for CSF1, c-fms, in osteoclasts results in a high bone mass phenotype, smaller osteoclasts *in vivo* and an impaired response to an anabolic PTH regimen

**DOI:** 10.1371/journal.pone.0247199

**Published:** 2021-02-19

**Authors:** Meiling Zhu, Ben-hua Sun, Erin Nevius, Jared Kaplan, João Pereira, Karl Insogna

**Affiliations:** 1 Departments of Medicine, Yale School of Medicine, New Haven, CT, United States of America; 2 Departments of Immunobiology, Yale School of Medicine, New Haven, CT, United States of America; Oklahoma State University, UNITED STATES

## Abstract

The receptor for Colony Stimulating Factor 1 (CSF1), c-fms, is highly expressed on mature osteoclasts suggesting a role for this cytokine in regulating the function of these cells. Consistent with this idea, *in vitro* studies have documented a variety of effects of CSF1 in mature osteoclasts. To better define the role of CSF1 in these cells, we conditionally deleted c-fms in osteoclasts (c-fms-OC^-/-^) by crossing c-fms^flox/flox^ mice with mice expressing Cre under the control of the cathepsin K promoter. The c-fms-OC^-/-^ mice were of normal weight and had normal tooth eruption. However, when quantified by DXA, bone mass was significantly higher in the spine and femur of female knock out mice and in the femurs of male knock out mice. MicroCT analyses of femurs showed that female c-fms-OC^-/-^ mice had significantly increased trabecular bone mass with a similar trend in males and both sexes demonstrated significantly increased trabecular number and reduced trabecular spacing. Histomorphometric analysis of the femoral trabecular bone compartment demonstrated a trend towards increased numbers of osteoclasts, +26% in Noc/BPm and +22% in OcS/BS in the k/o animals but this change was not significant. However, when the cellular volume of osteoclasts was quantified, the c-fms-OC^-/-^ cells were found to be significantly smaller than controls. Mature osteoclasts show a marked spreading response when exposed to CSF1 in a non-gradient fashion. However, osteoclasts freshly isolated from c-fms-OC^-/-^ mice had a near complete abrogation of this response. C-fms-OC^-/-^ mice treated with (1–34)hPTH 80 ng/kg/d in single daily subcutaneous doses for 29 days showed an attenuated anabolic response in trabecular bone compared to wild-type animals. Taken together, these data indicate an important non-redundant role for c-fms in regulating mature osteoclast function *in vivo*.

## Introduction

Colony Stimulating Factor-1 is one of two cytokines required for normal osteoclastogenesis. In its genetic absence, osteoclastogenesis fails, leading in mice to an osteopetrotic phenotype in which no osteoclasts are present in bone [1]. The role of CSF1 in osteoclastogenesis is incompletely understood but it appears to participate in early osteoclast differentiation as well as to play an important anti-apoptotic role in these cells. Thus, one of the earliest events in osteoclastogenesis is an upregulation in expression of the receptor for CSF1, c-fms, which is subsequently followed by upregulation of RANK [2]. It is likely that CSF1 engagement of c-fms plays a role in the initial expression of RANK on osteoclast precursors. CSF1 also induces expression of c-fos [3], which is absolutely required for normal osteoclastogenesis since in its genetic absence osteoclasts fail to form [4]. Several pro-survival pathways are regulated by CSF1 in osteoclasts, including the ubiquitination of the proapoptotic factor Bim [5] and stimulation of the electroneutral sodium bicarbonate exchanger [6].

The CSF1 receptor, c-fms is highly expressed in osteoclasts and immunohistochemical studies indicate that the mature osteoclast is one of the cells with the highest level of expression of this receptor *in vivo* in bone [7]. In addition to its anti-apoptotic actions, CSF1 has been shown to exert a variety of other effects on mature osteoclasts including rapid cytoskeletal remodeling and motility [8]. The molecular pathways that regulate CSF1 actions on the cytoskeleton of mature osteoclasts depend heavily on activation of small GTPases, most particularly Rac [9, 10]. Breast cancer associated gene 3 is a direct binding partner of Rac1 in mature osteoclasts and appears to modulate the cytoskeletal effects of Rac in these cells [11]. Importantly, inhibiting Rac has been shown to inhibit bone resorption [12]. Interestingly, there are three principal isoforms of Rac, but it appears that Rac2 mediates CSF1-dependant osteoclast motility [13]. Through a mechanism that remains incompletely understood, *in vitro* studies have demonstrated that CSF1 increases the size of mature osteoclasts [14]. In those experiments, the increase in size was accompanied by increased resorptive activity per cell since the number of osteoclasts did not increase.

Given the importance of CSF1 in skeletal metabolism and in osteoclast formation and function in particular, it is not surprising that germline deletion of c-fms results in a severe osteopetrotic phenotype in mice with a total lack of osteoclasts in bone [15]. However, this model provides no insights into the action of CSF1 in mature osteoclasts *in vivo*. Therefore, we selectively deleted c-fms in osteoclasts in an effort to better understand the contribution of CSF1 signaling in these cells to bone homeostasis *in vivo*.

## Materials and methods

### Materials

Recombinant human CSF1 was a generous gift from Genetics Institute (Cambridge, MA). Alpha-MEM cell culture media and fetal bovine serum were from Sigma (St. Louis, MO). Alexa Fluor 488 phalloidin was from Molecular Probes (Eugene, OR). Etched gridded coverslips were from BELLCO Glass, Inc. (Vineland, NJ). Vectashield mounting medium containing DAPI was from Vector Laboratories (Burlingame, CA). Anti-c-fms antibody was from Abcam® (Cambridge, MA). Hanks Buffered Salt Solution was from GIBCO (Carlsbad, CA). Recombinant murine CSF1 and murine Receptor Activator of NFκB Ligand (RANKL) were from R&D Systems (Minneapolis, MN). Human (1–34) PTH was from BACHEM Americas Inc. (Torrance, CA). Acid Phosphatase, Leukocyte was from Sigma-Aldrich, Inc. (St. Louis, MO).

### Animal care and husbandry

Animals were housed and maintained in AALC-approved animal care facilities in the Yale University School of Medicine. Animal husbandry is per the guidelines of the Yale Animal Resources Center. Animals are monitored daily by veterinarians and housed in cages that include no more than five animals. Mice were fed standard rodent chow from a commercial vendor. In addition to daily monitoring by veterinarians, the investigators monitored the animals on average twice a week. The study did not involve any procedures requiring anesthesia. Subcutaneous injections were given by habituating animals to being hand-held after which parathyroid hormone was administered subcutaneously. Sacrifice was accomplished by deep isoflurane anesthesia, followed by cervical dislocation. This study was approved by the Yale Institutional Care and Use Committee.

### Engineering mice with selective deletion of c-fms in osteoclasts (c-fms-OC^-/-^)

Mice in which both alleles of the c-fms gene are floxed were kindly provided to us by Dr. Jeffery Pollard, Albert Einstein College of Medicine. The mice are on a mixed genetic background 129-Ola/C57BL/6 [16]. Mice in which the cDNA for cathepsin K was knocked into the cathepsin K locus such that the expression of cre recombinase is under the control of the endogenous cathepsin K promoter, were kindly provided by Professor Shigeaki Kato, University of Tokyo, Tokyo, Japan. The Ctsk-Cre mice (CtskCre/+), originally on a hybrid C57BL/6 and CBA genetic background, were backcrossed for four generations onto a C57BL/6J background [17]. Breeding mice expressing cre under the control of the cathepsin K promoter with mice homozygous for the c-fms floxed allele resulted in mice expressing cre but heterozygous for the floxed c-fms allele. These mice were crossed back to mice homozygous for the floxed c-fms allele, resulting in mice with selective deletion of c-fms in mature osteoclasts (c-fms-OC^-/-)^. In this study, littermate mice homozygous for the c-fms floxed allele but not expressing cre (c-fms^flox/flox^) were used as a control. There have reports of expression of cathepsin K in gametes resulting in germline transmission [18]. However, we have previously reported that we have not found gamete expression of cre in these animals [19]. It has also been reported that osteocytes can, under certain circumstances express cathepsin K [20]. Since the current work reports change in bone mass in the c-fms-OC^-/-^ and since osteocytes regulate bone turnover, we also examined cre expression in those cells. There was no cre expression in osteocytes isolated from c-fms-OC^-/-^ mice (**S1 Fig**).

### Engineering mice expressing GFP in c-fms-OC^-/-^ osteoclasts

Transgenic mice engineered to express GFP only in cells expressing cre recombinase (B6.126(Cg)-Tg(CAG-Bgeo/GFP)21Lbe/J, stock number 004178) were purchased from Jackson laboratories. These animals were crossed to the c-fms-OC^flox/flox^ mice to yield mice with the genotype c-fms-OC^flox/+^/Tg^+/^ Cre^-/^ or c-fms-OC^-/+^/Tg^+/^ Cre^+/^. The c-fms-OC^flox/+^/Tg^+/^ Cre^-/^ animals were then crossed to the c-fms-OC^-/+^ Cre^+/^ mice. Animals with the genotype c-fms-OC^-/-^/Tg^+/^Cre^+/^ had osteoclasts that expressed GFP. Animals with the genotype c-fms-OC^+/+^/Tg^+/^Cre^+/^ were used as controls.

### Hematologic parameters

Whole blood was taken from 20 (10 control and 10 knock out) animals to measure hemoglobin and complete cell counts. These analyses were performed by ANTECH Diagnostics (Lake Success, NY).

### Osteoclast isolation and immunostaining

Mature osteoclasts from c-fms-OC^-/-^ and control mice were isolated from neonatal murine long bones by mechanical disaggregation as previously reported [8, 21] and plated onto gridded coverslips for 3 hours in α-MEM containing 10% FBS. The coverslips were washed with HBSS once. They were then incubated with anti-c-fms antibody for 1 hour. After three washes, the slides were incubated with goat anti-rabbit Alexa Fluor® 488 for 45 minutes. The cells were then fixed in 1% PFA for 15 min. All washes and incubations were performed on ice. Cells were examined using a Zeiss LSM 710 confocal imaging system.

### Osteoclast spreading assay

Mature osteoclasts were isolated from neonatal murine long bones and cultured as previously reported [8, 21]. Cells were imaged before and after 15 minutes of treatment with either 2.5 nM CSF1 or vehicle. Changes in cell area were quantified using ImageJ and expressed as percent change from baseline cell area as previously described [13]

### Osteoclast resorbing activity

Mature osteoclasts from c-fms-OC^-/-^ and from control mice were isolated from neonatal murine long bones as previously reported [8, 21]. The cells were plated in Osteo Assay® plates in α**-**MEM containing 10% FBS. 24 hours later, media were aspirated, and 600 μL of 10% sodium hypochlorite added to each well for 5 min at room temperature. The wells were then aspirated, washed twice with 900 μL of deionized water and allowed to dry completely at room temperature. Pit area was measured using Adobe Photoshop CS3 (Adobe Systems Inc., San Jose, Ca) and expressed as number of pixels.

### Bone density measurements

*In vivo* bone density measurements were using a PIXImus densitometer (Lunar, Madison, WI) as previously reported [22, 23], or with a Faxitron Ultrafocus densitometer (Faxitron-Hologic, Tucson, AZ) for some male mice (see **S4 Fig**).

### MicroCT analyses

Microcomputed tomography was performed as previously reported using a Scanco μCT-35 instrument (Scanco, Bruttisellen, Switzerland) [23].

### Bone histomorphometry

Femurs were removed, stripped of soft tissue, and fixed in 70% ethanol. They were then dehydrated through graded ethanol, cleared in toluene, infiltrated with increasing concentrations of methyl methacrylate, and embedded in methyl methacrylate according to previously described methods [22, 23]. Analyses were performed on 5-μM thick sections stained with toluidine blue, pH 3.7, using a Nikon microscope interfaced with the OsteoMeasure system software and hardware (Osteometrics, Atlanta, GA). Measurements were obtained in the distal femur in an area of cancellous bone that measured ∼2.5 mm^2^, containing only secondary spongiosa and located 0.5- and 2.5-mm proximal to the epiphyseal growth cartilage. All parameters were defined according to the American Society for Bone and Mineral Research histomorphometry nomenclature [24].

### Osteoclastogenesis assay

Bone marrow was harvested from knock out and control mice and cultured overnight in α-MEM containing 10% FBS and 100 ng/ml CSF1. The next morning, non-adherent cells were harvested, layered onto an equivalent volume of Ficoll-Hypaque and centrifuged at 700 X G for 20 min. Cells at the interface were collected, washed in PBS twice, and plated at a final concentration of 1.3x10^5^ cells/well in 24-well plates in α-MEM containing 10% FBS, 50 ng/mL CSF1 and 75 ng/mL RANKL. After 5 days in culture, with a media change every 2 days, cells were stained with an Acid Phosphatase, Leukocyte kit to identify cells positive for tartrate-resistant acid phosphatase. The number of cells was counted in a predetermined field. For each genotype between 49 and 50 fields were measured.

### PTH treatment protocol

Fifty-four animals (27 control and 27 knock out) were randomly assigned to treatment with single daily injections of either vehicle (10 mM acetic acid containing 2% heat-inactivated mouse serum) or 80 ng/g BW of h(1–34)PTH for 29 days as previously reported [22]. There were four treatment groups (vehicle/KO: 13 animals, PTH/KO: 14 animals, vehicle/control: 13 animals, PTH/control: 14 animals). Bone density was measured by DXA before and after 29 days of treatment.

#### Three-dimensional histology by two-photon microscopy

Three-dimensional histology using two-photon microscopy was performed using previously published methodology [25]. Femurs were cleaned of soft tissue, fixed in 4% PFA, rehydrated in 30% sucrose at 4°C and placed in Tissue Tek optimum cutting temperature compound (Sakura Inc., Torrance, CA). Samples were immediately frozen in EtOH and dry ice and stored at -80°C. Femur bone and marrow tissue were cut longitudinally with a cryostat to expose bone marrow cells and bone (~250μm) for 2-photon microscopy. 2-photon microscopy images were acquired using an Olympus BX61WI fluorescence microscope with a 20x, 0.95NA water immersion Olympus objective and dedicated single-beam LaVision TriM laser-scanning microscope (LaVision, Biotec), controlled by Imspector software. The metaphyseal region along the growth plate and individual trabeculae in the metaphysis region were scanned with femtosecond Chameleon Vision II titanium:sapphire (Coherent, Santa Clara, CA) laser pulses at 900 nm wavelength. The total imaging volume was 500x500x100 μm. At least 10 3-D images were analyzed per femur using Imaris software (Bitplane Inc, South Windsor, CT). Osteoclasts were visualized by GFP fluorescence intensity and anatomical position adjacent to bone as detected by second harmonic excitation. Quantification of osteoclast volumes was performed using Imaris (Bitplane Inc, South Windsor, CT) software, using the “surfaces” module to calculate osteoclast volume based on GFP fluorescence intensity.

### Statistical analyses

The two-sample Kolmogorov-Smirnov test was used to calculate the size distribution of osteoclasts in femurs of c-fms-OC^-/-^/Tg^+/^Cre^+/^ and c-fms-OC^+/+^/Tg^+/^Cre^+/^ mice. Unpaired two-tailed Student’s t-tests calculated using. GraphPad Prism v7.0 were used to calculate significance when appropriate, as indicated in the figure legends. Data presented as M±SEM. The error bars in figures reflect SEM. A value of p < 0.05 was considered to indicate a statistically significant difference.

## Results

### Deletion of c-fms in mature osteoclasts

**Fig 1:**
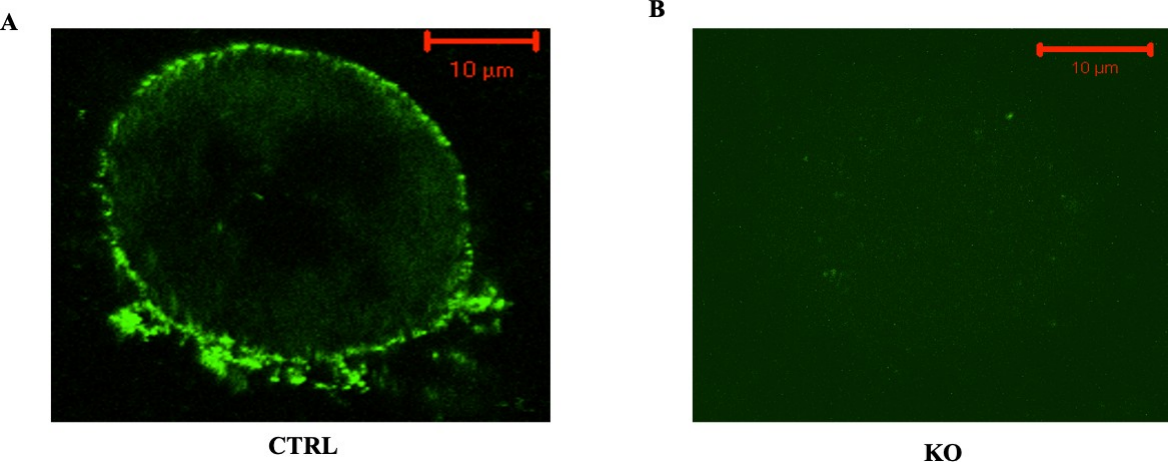
C-fms immunostaining of freshly isolated mature osteoclasts from control and c-fms-OC^-/-^ mice. To confirm deletion of c-fms in mature osteoclasts, immunostaining was performed on osteoclasts freshly isolated from control and knock out mice. (A) CTRL cell demonstrating intense c-fms membrane staining. (B) No staining was observed in the knock out cells. Findings were confirmed in 3 separate litters.

To confirm deletion of c-fms in mature osteoclasts, live cell immunostaining was performed on mature osteoclasts freshly isolated from control and knock out mice. Cell-surface expression of c-fms was observed in 68% of control osteoclasts (71 of 104) while staining was observed in only 12% of knock out osteoclasts (14 of 116), **Fig 1**. There was no staining observed when FITC-IgG was substituted for the primary antibody or when only secondary antibody was used for staining (**S2 Fig**). These findings were confirmed in 3 separate litters. The fact that there was c-fms expression in some knock out osteoclasts likely reflects the inherent limitations in any conditional knock out experiment, i.e. less than 100% deletion.

### Hematologic parameters are unchanged in c-fms-OC^-/-^ mice

Hematologic parameters were analyzed in ten 4–8 week old control and ten 4–8 week old knock out mice. As shown in **S1 Table**, blood hemoglobin concentration, total white blood cell count, as well as the numbers of lymphocytes and monocytes, were not significantly different between the two groups. Data for male and female animals are provided separately in **S3 Fig.**

### c-fms-OC^-/-^ mice have higher BMD

**Fig 2:**
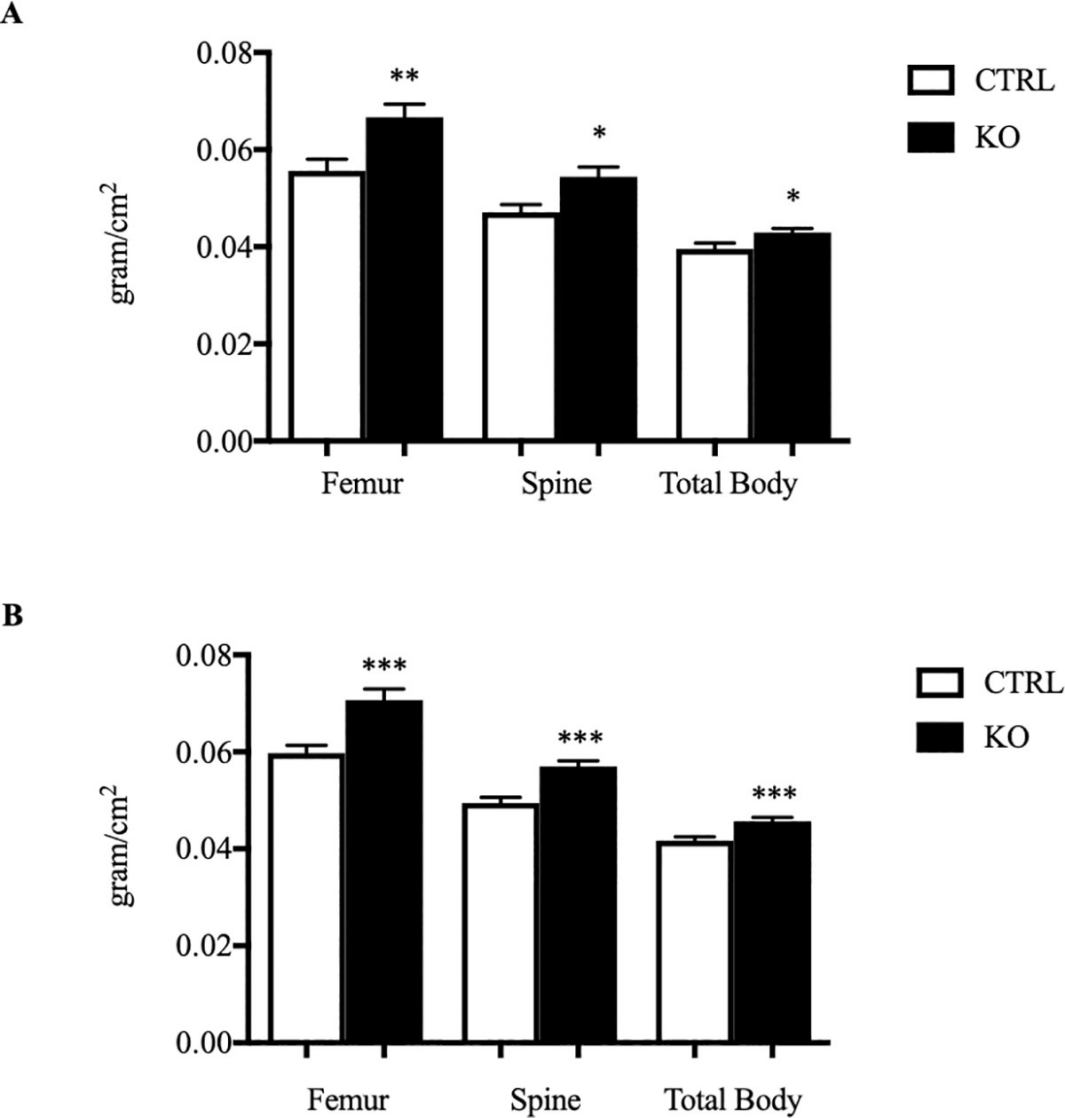
BMD determined by DXA in control and c-fms-OC^-/-^ mice. (A) DXA data in 6-week old CTRL and KO mice. CTRL n = 10 (3 male, 7 female). KO n = 12 (5 male, 7 female). (B) DXA data in 8-week old CTRL and KO mice. CTRL n = 21 (6 male, 15 female) and KO n = 27 (11 male, 16 female). *p < 0.05, **p < 0.01, ***p < 0.001, ****p < 0.0001. p-values were calculated using an unpaired two tailed Student’s t-test.

Bone density was assessed by DXA in 8-week old control (n = 21; 6 male, 15 female) and knock out (n = 27; 11 male, 16 female) mice. As shown in **Fig 2B**, bone density in the femur, spine, and total body were all significantly higher in the knock out animals. Femoral bone density was increased by 18% in the knock out animals compared to controls (0.0707±0.0020 vs. 0.0598±0.0016 g/cm^2^, p = 0.0005; KO vs. CTRL respectively). Spinal BMD was 15% higher compared to controls (0.0570±0.001 vs. 0.0495±0.0011 g/cm^2^, p < 0.0001). Total body BMD was 10% greater in the knock out animals (0.0457±0.001 vs. 0.0417±0.0008 g/cm^2^, p = 0.0006) as well. Bone density data in 6-week old animals demonstrated the same changes (**Fig 2A**). When bone density data were analyzed separately for male and female animals in 5–6 week old animals, the effect of deleting c-fms in osteoclasts appeared to be somewhat greater in female mice (**S4 Fig**). Female mice had significantly higher spinal and femur BMD. Male mice also had a significantly femur BMD and while there was a trend toward higher spinal BMD in the male mice, this change did not reach significance.

### MicroCT analyses demonstrate higher trabecular bone mass in c-fms-OC^-/-^ mice

**Fig 3:**
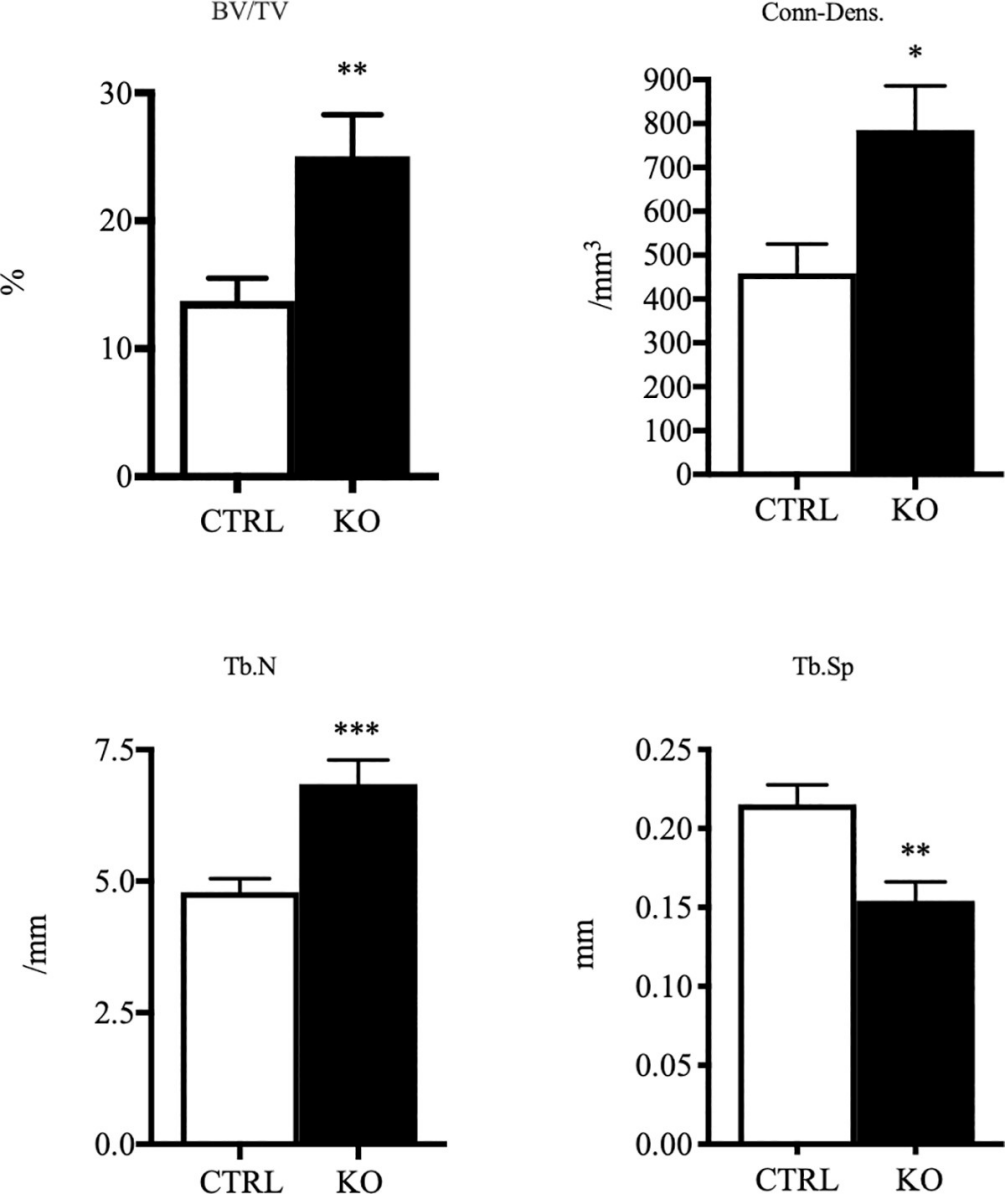
Increased trabecular bone mass in c-fms-OC^-/-^ mice. Micro-CT analysis of 5-6-week old CTRL and KO mice. CTRL n = 16 (8 male, 8 female) and KO n = 16 (8 male, 8 female). Significantly increased trabecular bone mass was observed in the KO mice. *p < 0.05, **p < 0.01, ***p < 0.001, p-values were calculated using an unpaired two tailed Student’s t-test.

To assess the effects of selective deletion of c-fms in osteoclasts on cortical and trabecular bone, microCT analyses were undertaken in 16 knock out (8 male, 8 female) and 16 control (8 male, 8 female) animals age 5-6-weeks. As shown in **Fig 3**, there was a marked increase in trabecular bone mass which was 82% higher in the knock out animals (KO: 25.1±3.2 vs. CTRL: 13.7±1.8%, p < 0.01). Consistent with this finding, trabecular number was 43% higher (KO: 6.846±0.459 vs. CTRL: 4.790±0.256 /mm, p < 0.001), connectivity density was 71% greater (KO: 785.5±100.7 vs. CTRL: 459±66.76 /mm^3^, p < 0.05) and trabecular spacing was 28% lower (KO: 0.154±0.012 vs CTRL: 0.215±0.012 mm, p < 0.01) in the knock out animals. When data from male and female animals were analyzed separately (**S5 and S6 Figs)**, there again appeared to be slightly greater effect in female animals. Thus, while both sexes demonstrated an increase in trabecular BV/TV the change was only significant in the female animals. However, both sexes showed a significant increase in trabecular number and a significant decrease in trabecular spacing. In contrast to the findings in the trabecular compartment, cortical bone volume and cortical thickness were unaffected (**S7 Fig**).

### Histomorphometric analyses in femoral trabecular bone of c-fms-OC^-/-^ mice

**Table 1** summarizes the cellular parameters in trabecular bone of 7 c-fms-OC^-/-^ and 8 control mice 5-6-weeks old. Although there were slight increases in the number of osteoclasts (NOc/BPM, +26%) as well as in osteoclast surface (OcS/BS, +22%), as well as slight decreases in the number of osteoblasts (Nob/BPm, -24%) and in osteoblast surface (ObS/BS, -25%) in c-fms-OC^-/-^ mice, none of these changes were significant. Data for male and female animals are provided separately in **S8** and **S9 Figs.**

**Table 1:** Histomorphometric analysis of the femoral trabecular bone compartment in c-fms-OC^-/-^ mice.

Genotype	NOc/BPm (/mm)	OcS/BS (%)	NOb/BPm (/mm)	ObS/BS (%)
**CTRL**	2.15 ± 0.5	7.27 ± 1.5	29.8 ± 3.9	28.9 ± 3.8
**KO**	2.70 ± 0.4	8.9 ± 1.47	22.8 ± 4.4	21. 8 ± 4.0
**P value**	0.4	0.5	0.3	0.2

### Reduced volume of mature osteoclasts in the proximal metaphyseal region of the femur in c-fms-OC^-/-^ mice

**Fig 4:**
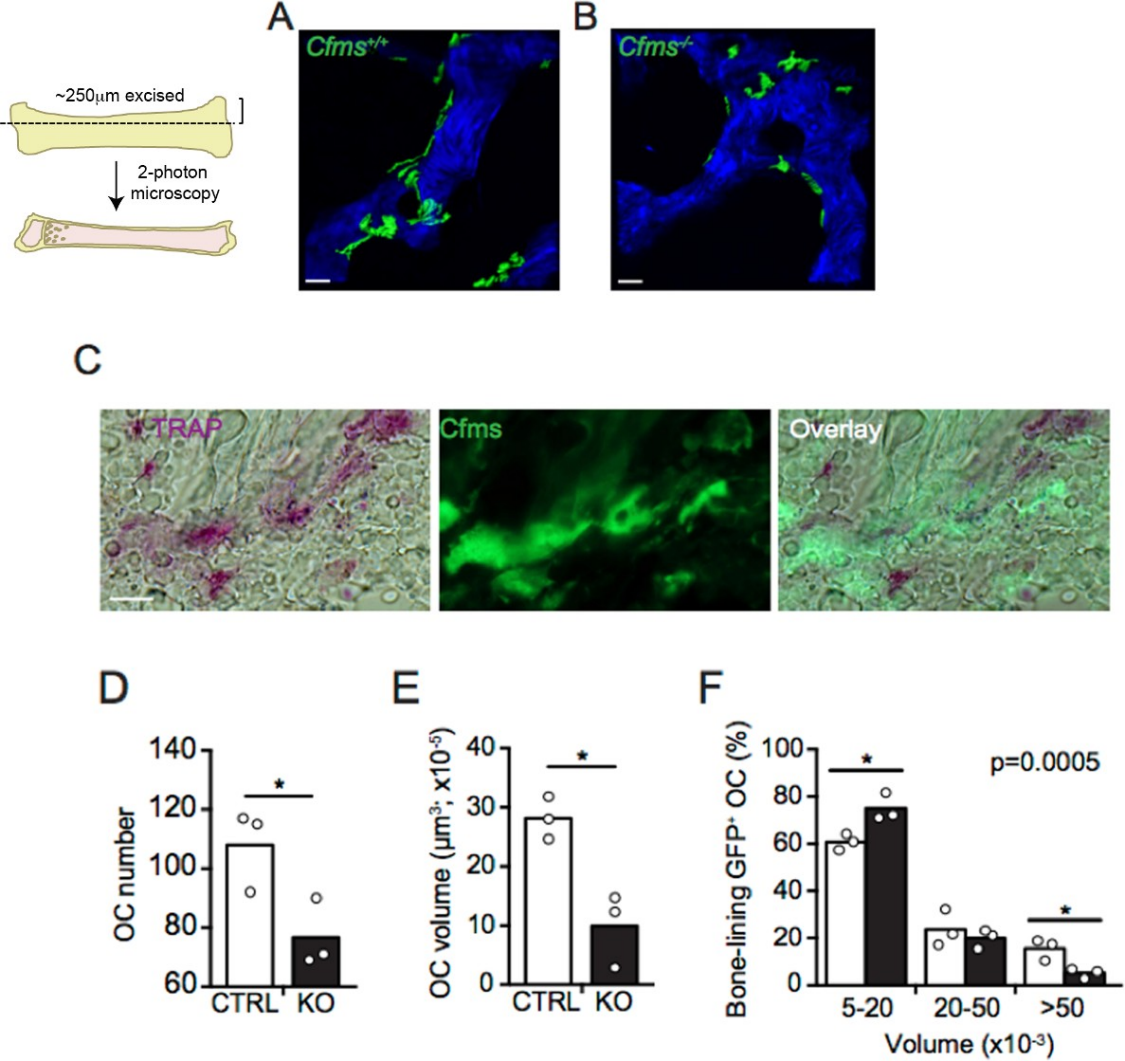
*In vivo* quantification of osteoclast number and mass in control and c-fms-OC^-/-^ mice. Two-photon microscopy of 3-dimensional osteoclast/trabecular bone network in (A) CTRL and (B) KO mice. Bone matrix is depicted in blue (generated by 2^nd^ harmonic fluorescence detection of collagen) and GFP^+^ osteoclasts are depicted in green using Imaris isosurface software tool. (The image to the left of A shows how femurs were sectioned for the analyses.) Scale bar = 50 mm. Imaging volume is 500x500x100μm (original magnification 20x). (C) 7μm thick sections from CTRL mice were stained for TRAP (left) with chromogenic substrates (purple). Green signal shows c-fms expression in large cells lining trabecular bone (middle). Images were visualized by light (left panel) and fluorescent (middle panel) microscopy (original magnification 40x). Right panel depicts overlay. Scale bar = 50 μM. (D) Enumeration of osteoclasts and (E) quantification of total osteoclast mass in femurs of CTRL (white bar) and KO (back bar) mice using 2-photon microscopy images as in A and B. Bars indicate the mean and circles depict total osteoclast number in D and total osteoclast mass in mm^3^ in E from individual mice quantified from 10 2-photon images per mouse. (F) Size distribution of osteoclasts in femurs of CTRL (white bars) and KO (black bars) mice in the size (mm^3^) categories as indicated. Two-sample Kolmogorov-Smirnov test; p = 0.0005. Data is pooled from 3 independent experiments. *p < 0.05 unpaired two tailed Student’s *t*-test.

The increased bone mass in the c-fms-OC^-/-^ mice without a change in osteoclast number suggests a defect in the resorptive activity of the mature osteoclasts. Since osteoclast size is correlated with resorptive activity and since CSF1 has been shown to regulate osteoclast size [14] we wondered if there was any difference in the size of osteoclasts in the genetic absence of c-fms in these cells. Two-photon microscopy was used to assess the mean cell volume of osteoclasts *in vivo* in the metaphysis of control and knock out mice. As shown in **Fig 4A** and **4B**, osteoclasts apposed to bone could be identified both in the control and knock out mice. That these GFP positive cells were osteoclasts was confirmed by double staining for TRAcP (**Fig 4C**). However, there was a striking reduction in the mean cell number and volume of the c-fms-OC^-/-^ osteoclasts. The knock out osteoclasts were only a third as large as control cells (**Fig 4D and 4E**). This discrepancy in cell size was further analyzed by assessing the relative proportion of cells with cell volumes between 5–20 μm^3^, 20–50 μm^3^, and > 50 μm^3^. As seen in **Fig 4F** there were significantly more knock out osteoclasts between 5–20 μm^3^ in size and significantly fewer c-fms-OC^-/-^ > 50 μm^3^ in size, compared to the size distribution observed in control cells.

### Osteoclasts freshly isolated from c-fms-OC^-/-^ mice have a markedly impaired cytoplasmic spreading response to CSF1

**Fig 5:**
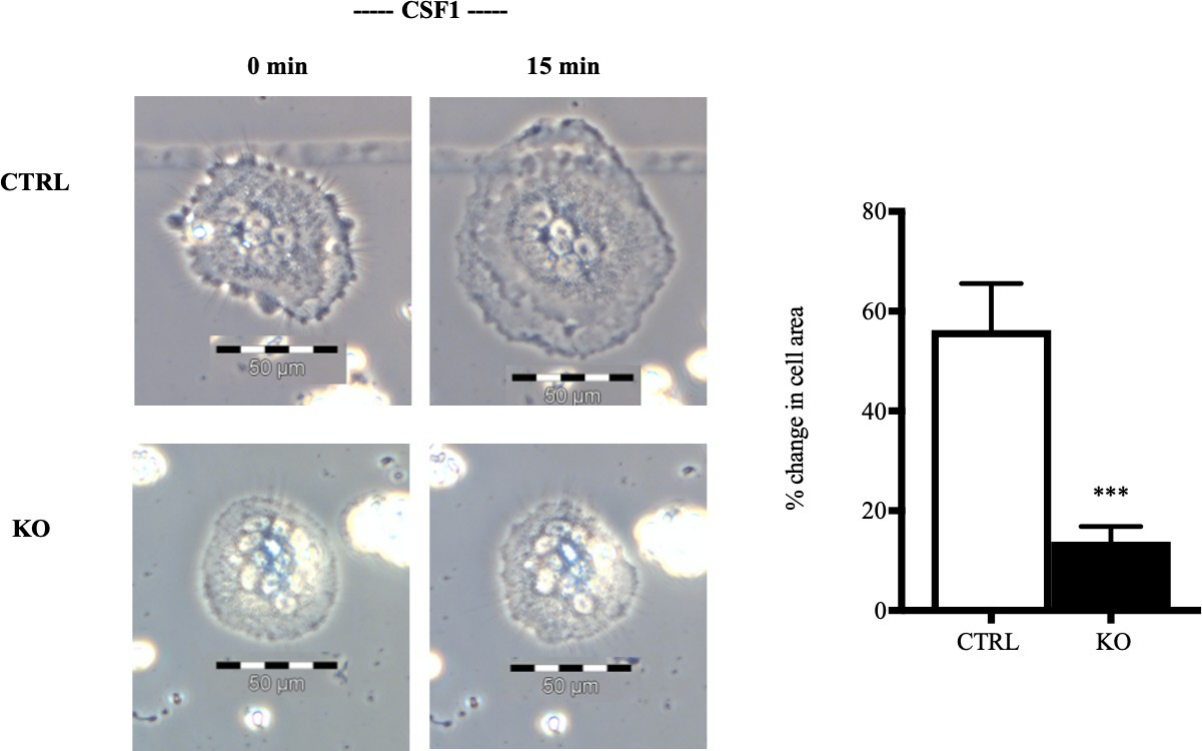
Inability of CSF1 to induce cytoskeletal remodeling in freshly isolated mature osteoclasts from c-fms-OC^-/-^ mice. In response to 15 min of treatment with 2.5 nM CSF1, the mean area of CTRL cells increased by 56% (n = 31) shown in top left image whereas the KO cells showed only a 14% change (n = 29) shown in bottom left image, a difference that was highly significant (***p < 0.001) shown in the plot. Scale bar = 50 μM. p-values were calculated using an unpaired two tailed Student’s t-test.

A consistent response to c-fms activation in osteoclasts is rapid cytoplasmic spreading [8, 21]. As shown in **Fig 5**, osteoclasts isolated from control animals had a normal spreading response increasing cells area by 56±9% (n = 31). In contrast, spread area increased by only 14±3% (n = 29) in osteoclasts freshly isolated from c-fms-OC^-/-^ mice. The difference in the response to CSF1 based on genotype was highly significant (p < 0.001).

### Bone marrow osteoclastogenesis is normal in c-fms-OC^-/-^ mice

**Fig 6:**
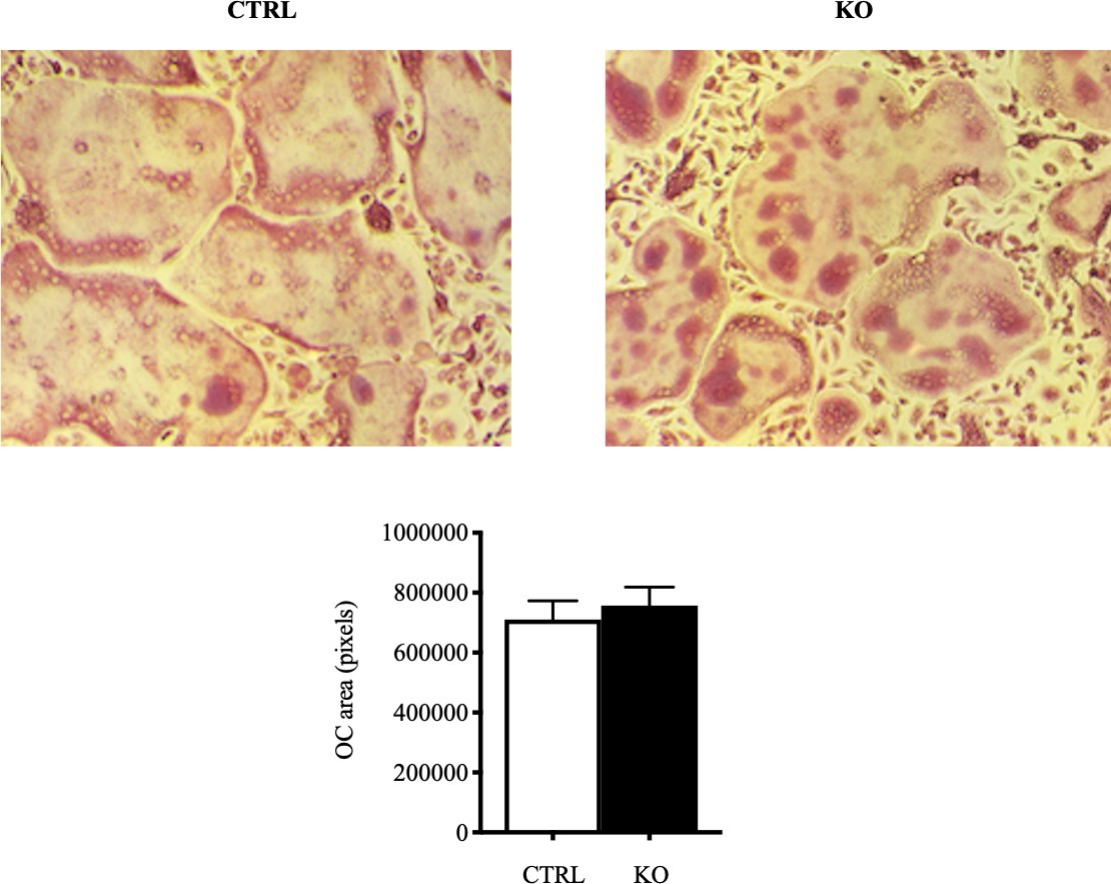
*In vitro* osteoclastogenesis using bone marrow precursors isolated from 8-week old male control and c-fms-OC^-/-^ mice. Osteoclastogenesis was unimpaired in KO mice as shown in the images and there was no difference in the total area of mature osteoclasts formed as quantified by TRAP staining based on genotype.

Since c-fms was deleted only from mature osteoclasts in the c-fms-OC^-/-^ mice, it was anticipated that there would be little effect on *in vitro* osteoclastogenesis. As shown in **Fig** 6, osteoclastogenesis was unimpaired as assessed qualitatively by TRAP staining of bone marrow precursor cells driven to form mature osteoclasts as well as quantitatively by measuring the total area of mature osteoclasts formed *in vitro*.

### Resorbing activity is impaired in mature c-fms-OC^-/-^ osteoclasts

**Fig 7:**
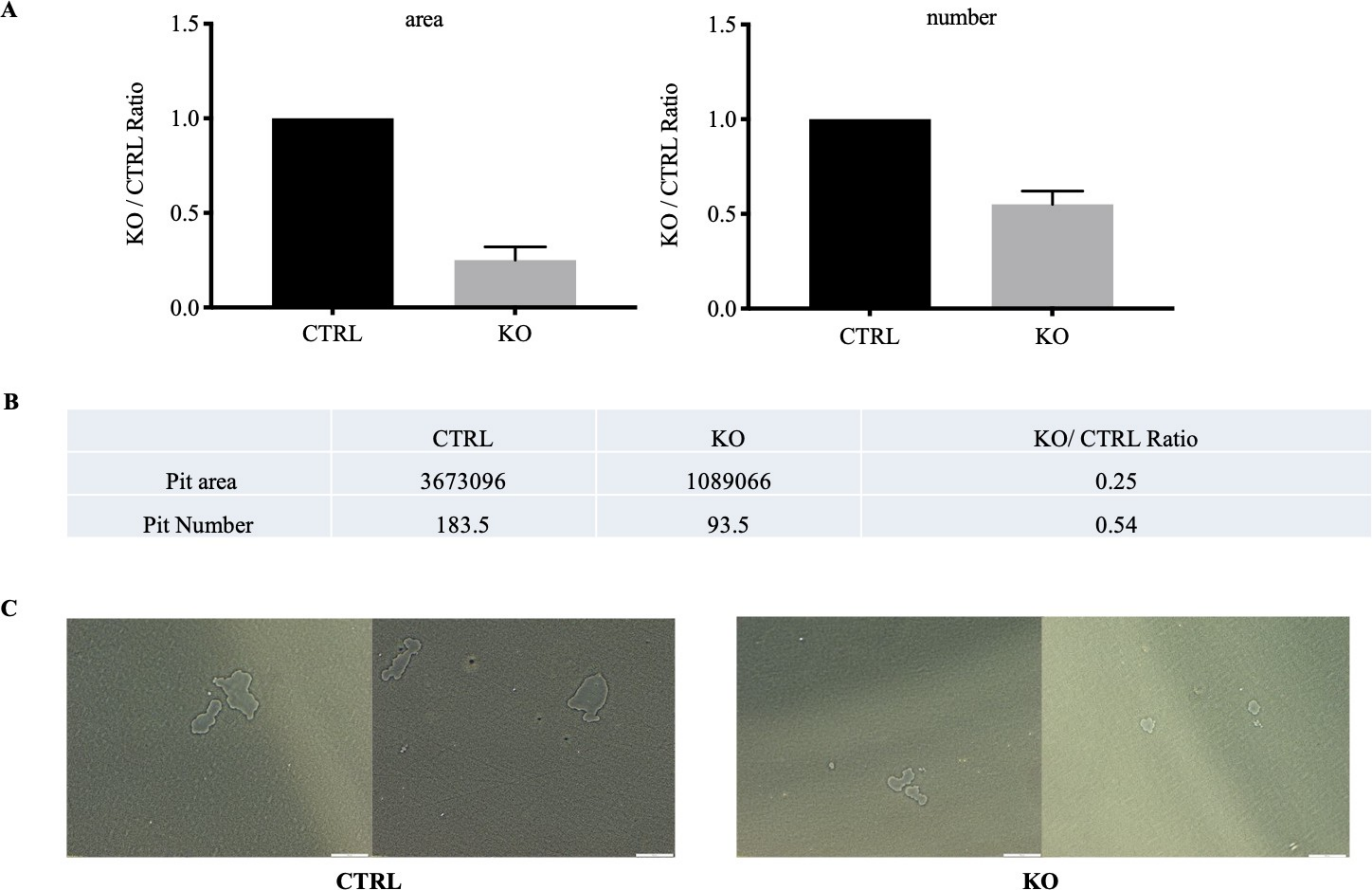
Impaired resorptive activity of c-fms-OC^-/-^ osteoclasts. (A) Area resorbed by authentic mature osteoclasts freshly isolated from control and KO mice. The area resobed by control cells was set a 100%. The area resorbed by osteoclasts was compared to that average number. (B) Number or resorption pits. The number of pits generated by CTRL osteoclasts was set to 100% and the number generated by KO cells compared to that value. The unit of measure is pixels. (C) Photograph of resorption pits created by CTRL (left two panels) and KO (right two) freshly isolated mature osteoclasts. Scale bar = 100 μM.

As noted in the Introduction, CSF1 increases osteoclast size and resorptive activity and the findings that c-fms-OC^-/-^ osteoclasts are significantly smaller than control cells is consistent with that. We therefore next determined whether the c-fms-OC^-/-^ osteoclasts also had impaired resorptive activity. As shown in **Fig** 7 mature osteoclasts isolated from c-fms-OC^-/-^ mice had impaired resorbing activity.

### Mice in which c-fms is deleted in osteoclasts have an attenuated response to an anabolic PTH regimen

**Fig 8:**
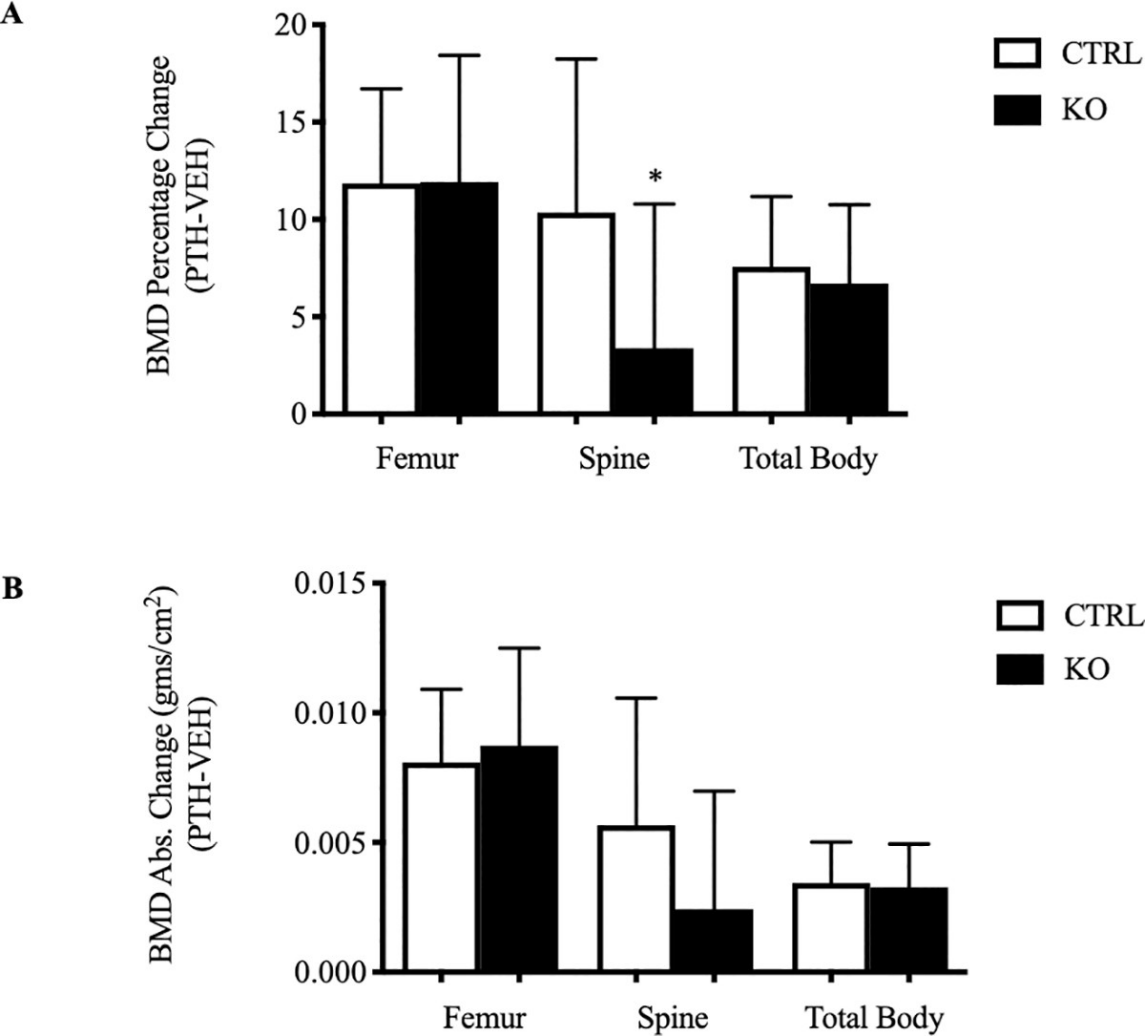
Attenuated anabolic response to PTH in c-fms-OC^-/-^ mice. (A) Percent change in BMD from baseline in 9-week old CTRL (open-bars) and KO mice (solid-bars) treated for 29-days with an anabolic PTH regime. (B) Absolute change in BMD (gms/cm^2^) with PTH in the two groups. The summarized data represent changes after subtracting the mean change in vehicle-treated animals of the corrosponding genotype. (CTRL: n = 14, 7 male, 7 female; KO: n = 14, 6 male, 8 female). *p = 0.02. p-values were calculated using an unpaired two tailed Student’s t-test.

It has been previously reported that PTH stimulates the production of CSF1 by osteoblasts and that this plays a role in PTH-induced bone resorption [7, 26]. It is now apparent that osteoclasts in addition to their critical role in mediating resorption, likely also contribute to bone anabolism by directly elaborating clastokines that promote osteoblast formation and function. We, therefore, wondered if the absence of c-fms in osteoclasts would alter the anabolic response to PTH. Specifically, we hypothesized that there might be a blunted resorptive response to the hormone when given once daily, which would be reflected in a more robust increase in bone mass. Conversely, it is possible that CSF1-mediated anabolic clastokine production would be abrogated, which would attenuate the anabolic response to PTH. As shown in **Fig 8**, when compared to the response in control animals, there was an attenuated anabolic response to PTH in the c-fms-OC^-/-^ mice at 9-weeks old, specifically in the spine. The spine is a skeletal envelope that is enriched with trabecular bone and the anabolic response to PTH is generally most robust in the spine. The increase in spinal BMD in response to PTH was nearly three times greater in the control mice as compared to mice lacking c-fms expression in mature osteoclasts (10.4±2.1 vs 3.4±2.0%, p = 0.02; CTRL vs KO, respectively). There were no significant differences in the PTH-induced increases in femoral and total body BMD based on genotype. The changes in absolute BMD values mirrored the findings when BMD was analyzed as percent change, but because of the variability in absolute BMD values, the change in absolute BMD at the spine did not reach statistical significance. Data summarizing the anabolic response to PTH in male and female animals are separately provided in **S10** and **S11 Figs**.

## Discussion

The principal finding of this paper is that c-fms, expressed in mature osteoclasts, has an important role *in vivo*. The genetic absence of c-fms in these cells resulted in an increased bone mass as measured by dual-energy absorptiometry. MicroCT analysis demonstrated that this was principally due to an increase in trabecular bone mass. Cortical bone mass was unaltered. The effect of deleting c-fms in osteoclasts on bone mass was more pronounced in female animals. Histomorphometric analyses showed no significant differences in osteoblast number or parameters of osteoblast function. Osteoclast numbers were not reduced. In fact, although not significant, there was a trend toward increased numbers of osteoclasts on bone surfaces. The mechanism of the increased bone mass, therefore, was not due to a defect in osteoclastogenesis. This is further supported by the normal ability of osteoclast precursors isolated from c-fms-OC^-/-^ mice to develop into mature osteoclast-like cells (**Fig 6**). These findings are in contrast to those in mice in which c-fms is deleted in the germline. These animals completely lack osteoclasts in bone and have an osteopetrotic phenotype [15]. Our data indicate that expression of c-fms late in the osteoclast lineage is not required for the formation of osteoclasts, but nonetheless does have an important role in the function of mature osteoclasts. Consistent with that, cells with the highest level of expression of c-fms in bone are, in fact, mature osteoclasts. CSF1 has been shown to have a wide variety of actions in mature osteoclasts including cytoskeletal remodeling, cell motility, cell survival, and gene transcription [3, 6, 13, 21]. Among the most dramatic and rapid of these effects is the appearance of large lamellipodia within minutes of exposing mature osteoclasts to CSF1 in a non-gradient manner [8]. As expected, in the genetic absence of c-fms, mature osteoclasts freshly isolated from c-fms-OC^-/-^ mice showed a markedly diminished spreading response to CSF1, which was more than five-fold less than that seen in control cells (**Fig 5**). The fact that there was any response at all likely reflects the fact that conditional deletion of c-fms was perhaps not achieved in every single mature osteoclast. However, our model was quite successful in deleting c-fms in the vast majority of mature osteoclasts, as documented by the absence of c-fms cell-surface immunostaining in the c-fms-OC^-/-^ cells.

In addition to abrogated cytoskeletal remodeling in the genetic absence of c-fms in mature osteoclasts, these cells were significantly smaller than control cells. Lees et al. have reported that exposing mature rabbit osteoclasts to CSF1 for 48 hours results in larger cells with greater resorption efficiency [14]. The investigators also noted that in general, larger osteoclasts are more efficient at resorbing bone than smaller osteoclasts. We used two-photon absorptiometry to quantify osteoclast volume in metaphyseal osteoclasts on bone. As summarized in **Fig 4**, there was a dramatic reduction in osteoclast volume consistent with these previous *in vitro* studies and supportive of the conclusion that the net resorptive capacity of these cells would be diminished. Our data demonstrating impaired resorptive activity in mature osteoclasts freshly isolated from the OC-c-fms^-/-^ mice (**Fig** 7) is entirely consistent with the work of Lees et al. and our own *in vivo* data. Since an impaired osteoclast resorptive response might be of therapeutic benefit in maximizing anabolic therapeutic regimens for low bone mass, we next determined whether the response to single daily PTH administration would be altered in these animals. We have previously reported that the anabolic response to PTH was augmented in mice in which osteoclast resorptive activity was impaired [22]. In particular, we showed that in the genetic absence of Rac2, basal resorptive activity was diminished in mature osteoclasts, albeit their numbers *in vivo* were actually normal-to-high [13]. We postulated the exaggerated anabolic response to PTH in those animals reflected the combined effects of an attenuated resorptive response to PTH without an alteration in the anabolic signal. In an analogous fashion, we wondered whether the absence of CSF1 would result in a diminished resorptive response to single daily PTH administration with an unaltered anabolic signal. However, contrary to our expectations, the anabolic response to PTH was significantly attenuated in the spine of female animals. The spine is a site enriched in trabecular bone and trabecular bone is well recognized to be the skeletal envelope where the anabolic response to intermittent PTH is most robust. Consequently, it is reasonable to expect an attenuated anabolic signal to be most evident in trabecular bone. The fact that the impaired response to PTH was most evident in female mice (**S10B** and **S11B** Figs), is of particular interest since women are at greatest risk for osteoporosis and an anabolic PTH treatment regimen is an approved treatment for that disease. Whether estrogen modifies the cellular actions of CSF1 is not known be we have not found differences in circulating levels of CSF1 in postmenopausal women treated with estrogen compared to postmenopausal estrogen-deficient women [27].

The mechanism by which c-fms could participate in mediating the anabolic signal to PTH is not directly addressed by this work; although, a working hypothesis is that its effect is mediated by the ligand for c-fms, CSF1 acting on osteoclasts. Consistent with this, we have shown that CSF1 is the principle colony stimulating activity released by osteoblasts in response to PTH [7]. We have shown that CSF1 induces transcription of the rate limiting enzyme for production of sphingosine-1-phosphate, sphingosine kinase 1, through a novel cis element in the 5’ region of the gene [28]. Sphingosine-1-phosphate is a known anabolic clastokine [29, 30]. Therefore, it is possible that PTH stimulates CSF1 production by osteoblasts that is released and subsequently engages c-fms on mature osteoclasts to stimulate productions of sphingosine-1-phosphate by activating sphingosine kinase 1. However, it is possible that CSF1 has direct anabolic actions in bone since it has been previously reported that administration of CSF1 *in vivo*, albeit in high doses, results in an increase in trabecular bone mass [31].

In summary, this study identifies an important role for c-fms in mature osteoclasts. Its genetic absence *in vivo* results in a high bone mass phenotype and a reduction in osteoclast size *in vivo*. Further, it suggests a potential role for this receptor in mediating PTH-induced bone anabolism.

## Supporting information

S1 Fig Cre is not expressed in osetoctyes of c-fms-Oc^-/-^ mice. **Upper panel:** Western blot for cre in whole cell lysates from two different KO osteclast preparations, lanes 1 and 2 (BM-OCL), and lysates prepared from osteocytes (Octy) cultured from KO mice. Cre was detected in both preparations of knock out osteoclasts but cre was not detected in osteocytes isolated from knock out mice. **Lower panel:** The blot shown in the upper panel was stripped and reprobed for β-actin to confirm equal protein loading in each lane.
(TIF)

S2 Fig C-fms immunostaining of freshly isolated mature osteoclasts from control mice. **A:** No staining was observed in cells isolated from control mice when FITC-IgG was substituted for the primary antibody, or **B:** when only secondary antibody was used for staining.
(TIF)

S3 Fig Hematologic parameters separately analyzed in 4–8 week old mice. **A:** male mice **B:** female mice. Male: CTRL: n = 5; KO: n = 5. Female CTRL: n = 5; KO: n = 5. Hematologic parameters were not statistically significant in either sex based on genotype.
(TIF)

S4 Fig DXA BMD separately analyzed in male and female 5–6 week old mice. **A:** male mice; CTRL n = 8; KO n = 8. Two cohorts of knock out male mice and littermate controls were studied. In between the first and second cohort, the DXA instrument used to measure BMD was changed from a PIXimus machine to a Faxitron Ultrafocus densitometer. The absolute BMD measurements differed on each machine. In neither cohort were there significant differences between control and knock out mice but the numbers of animals in each cohort was relatively small. To combine the data from the two cohorts, the control data from both cohorts for each site were combined and an average BMD calculated. The individual control data were then compared to this average value to generate a M±SEM which *per force* resulted in a mean value of 1.0. The indivual BMD data from the knock out male mice were then compared to the average absolute value for the control mice to generate a fold-change value. The M±SEM fold-change for each skeletal siteis summarized in A. **B:** DXA BMD by PIXimus in female mice; CTRL n = 8; KO n = 8. * p <0.05. p-values were calculated using an unpaired two-tailed Student’s t-test.
(TIF)

S5 Fig MicroCT analysis of femoral trabecular bone in 5–6 week old male mice. CTRL n = 8; KO n = 8. * = p<0.05. p-values were calculated using an unpaired two-tailed Student’s t-test.
(TIF)

S6 Fig MicroCT analysis of femoral trabecular bone in 5–6 week old female mice. CTRL n = 8; KO n = 8. * = p<0.05. ** = p<0.01. p-values were calculated using an unpaired two-tailed Student’s t-test.
(TIF)

S7 Fig A: MicroCT analysis of femoral cortical bone in 5–6 week old male mice. CTRL n = 8; KO n = 8. B: MicroCT analysis of femoral cortical bone in 5–6 week old female mice. CTRL n = 8; KO n = 8.
(TIF)

S8 Fig Histomorphometric analysis in 5–6 week old male mice. CTRL n = 4; KO n = 3.
(TIF)

S9 Fig Histomorphometric analysis in 5–6 week old female mice. CTRL n = 4; KO n = 4.
(TIF)

S10 Fig Response to an anabolic PTH regimen in 9 week old mice. **A:** male mice; CTRL n = 7; KO n = 6. **B:** female mice; CTRL n = 7; KO n = 8. * = p<0.05. p-values were calculated using an unpaired two tailed Student’s t-test.
(TIF)

S11 Fig Absolute changes in BMD in response to the anabolic PTH regimen in 9 week old mice. **A:** male mice; CTRL n = 7; KO n = 6. **B:** female mice, CTRL n = 7; KO n = 8.
(TIF)

S1 Table Hematologic parameters in 4–8 week old c-fms-OC^-/-^ mice. (DOCX)
