# Supplementary figures and images for: Selective deletion of the receptor for CSF1, c-fms, in osteoclasts results in a high bone mass phenotype, smaller osteoclasts in vivo and an impaired response to an anabolic PTH regimen
Source: PLoS One. 2021 Feb 19;16(2):e0247199. doi: 10.1371/journal.pone.0247199 (PMC7895546; doi:10.1371/journal.pone.0247199)

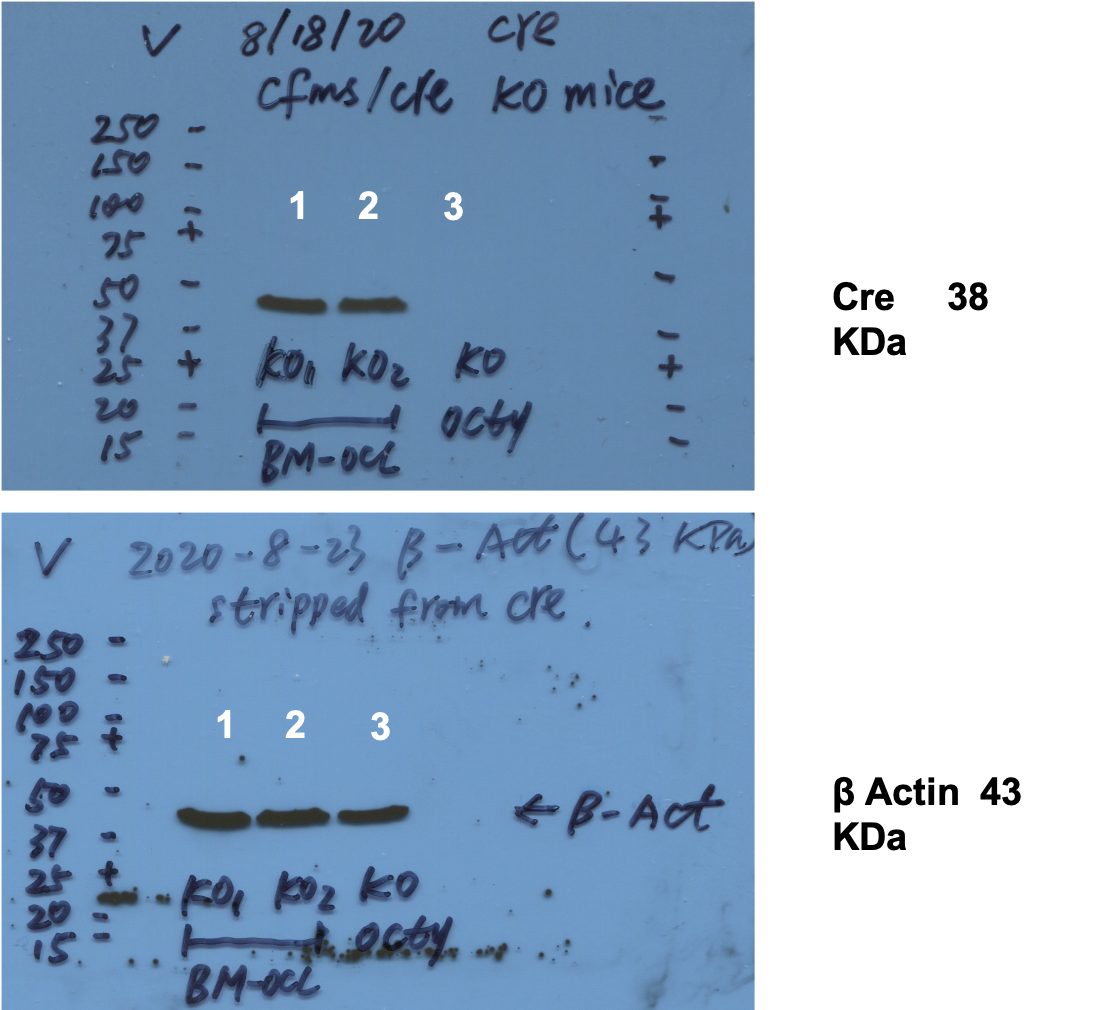

Supplement: S1 Fig — Upper panel: Western blot for cre in whole cell lysates from two different KO osteclast preparations, lanes 1 and 2 (BM-OCL), and lysates prepared from osteocytes (Octy) cultured from KO mice. Cre was detected in both preparations of knock out osteoclasts but cre was not detected in osteocytes isolated from knock out mice. Lower panel: The blot shown in the upper panel was stripped and reprobed for β-actin to confirm equal protein loading in each lane. (TIF) [file pone.0247199.s001.tif]

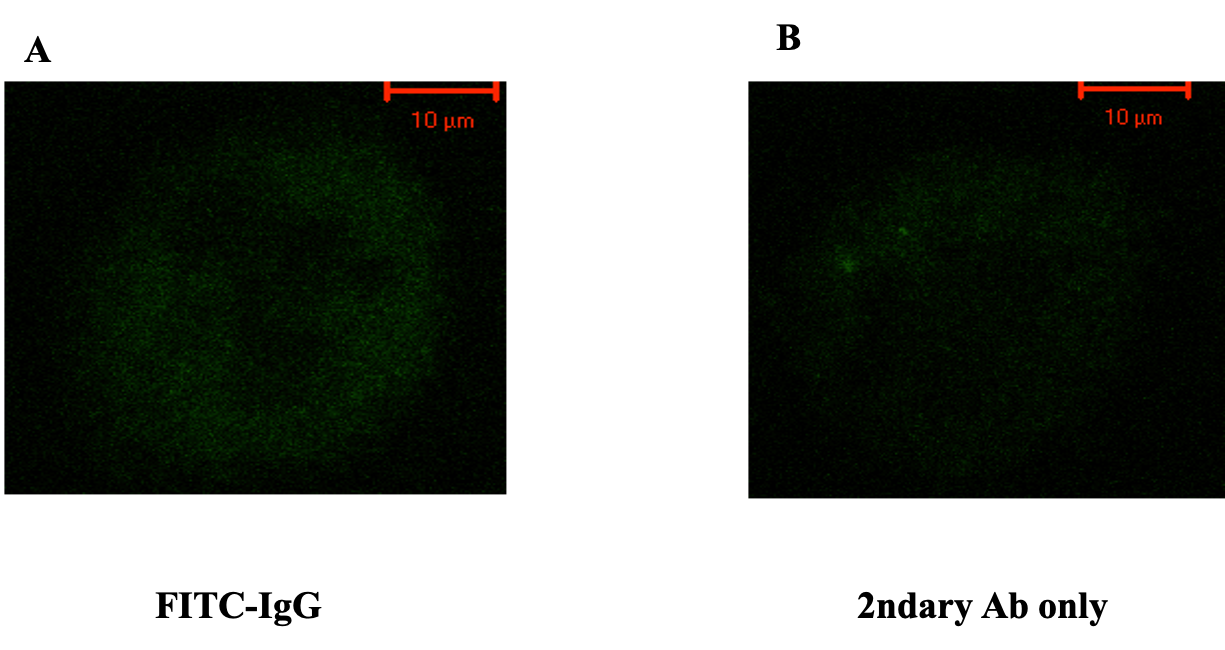

Supplement: S2 Fig — A: No staining was observed in cells isolated from control mice when FITC-IgG was substituted for the primary antibody, or B: when only secondary antibody was used for staining. (TIF) [file pone.0247199.s002.tif]

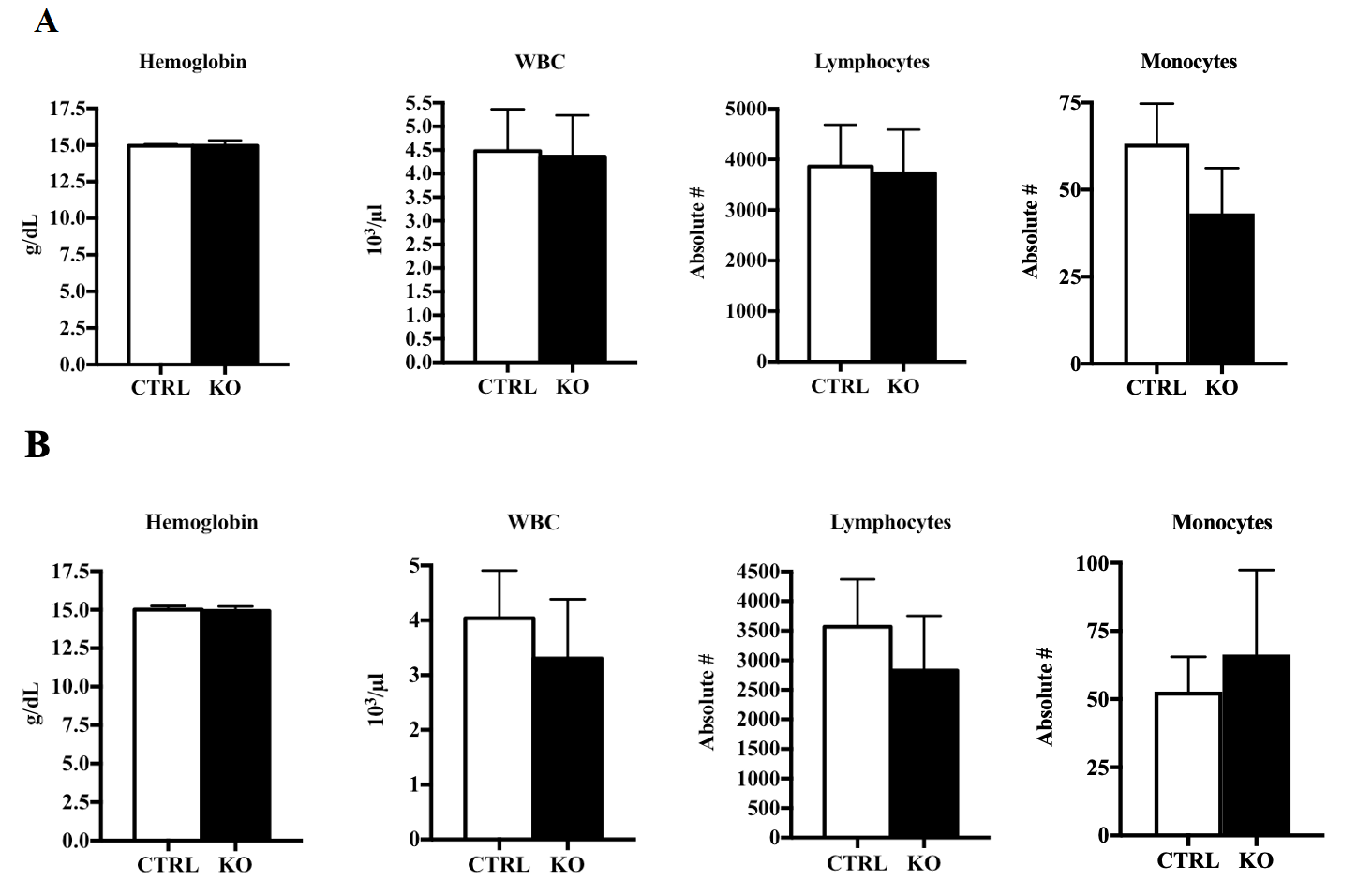

Supplement: S3 Fig — A: male mice B: female mice. Male: CTRL: n = 5; KO: n = 5. Female CTRL: n = 5; KO: n = 5. Hematologic parameters were not statistically significant in either sex based on genotype. (TIF) [file pone.0247199.s003.tif]

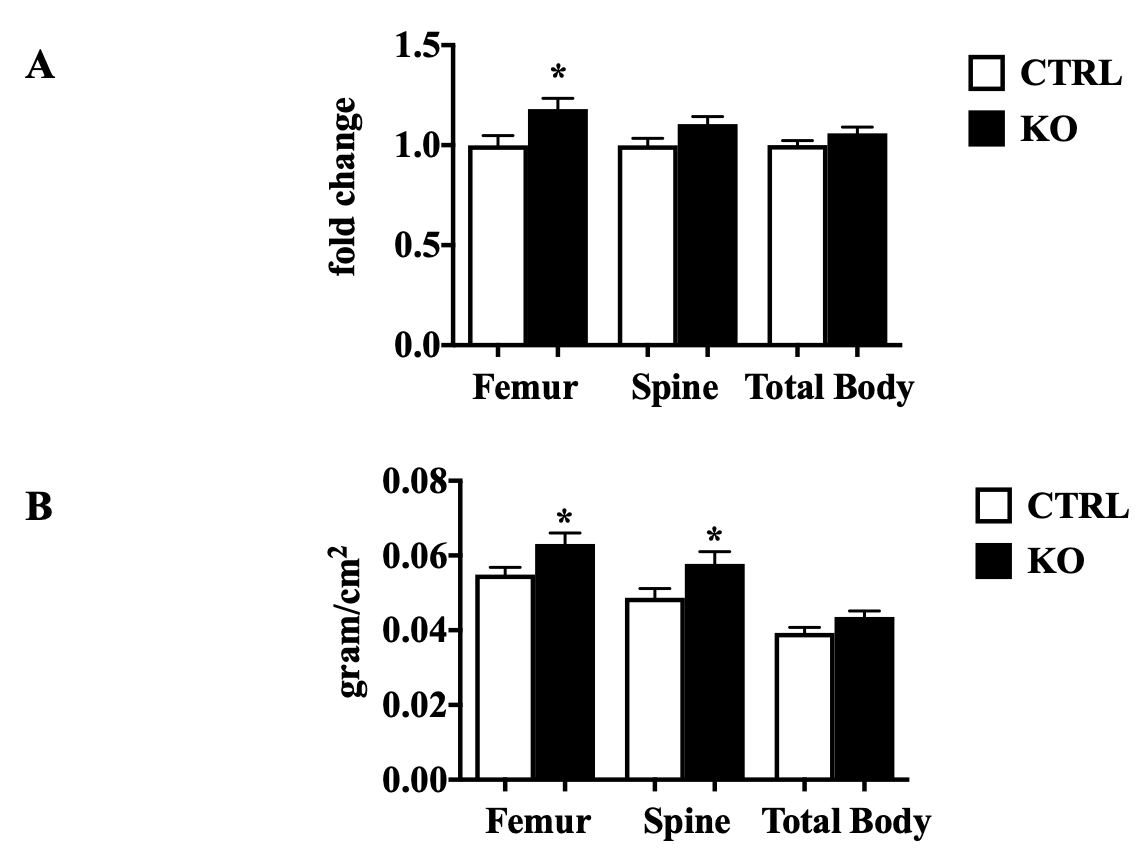

Supplement: S4 Fig — A: male mice; CTRL n = 8; KO n = 8. Two cohorts of knock out male mice and littermate controls were studied. In between the first and second cohort, the DXA instrument used to measure BMD was changed from a PIXimus machine to a Faxitron Ultrafocus densitometer. The absolute BMD measurements differed on each machine. In neither cohort were there significant differences between control and knock out mice but the numbers of animals in each cohort was relatively small. To combine the data from the two cohorts, the control data from both cohorts for each site were combined and an average BMD calculated. The individual control data were then compared to this average value to generate a M±SEM which per force resulted in a mean value of 1.0. The indivual BMD data from the knock out male mice were then compared to the average absolute value for the control mice to generate a fold-change value. The M±SEM fold-change for each skeletal siteis summarized in A. B: DXA BMD by PIXimus in female mice; CTRL n = 8; KO n = 8. * p <0.05. p-values were calculated using an unpaired two-tailed Student’s t-test. (TIF) [file pone.0247199.s004.tif]

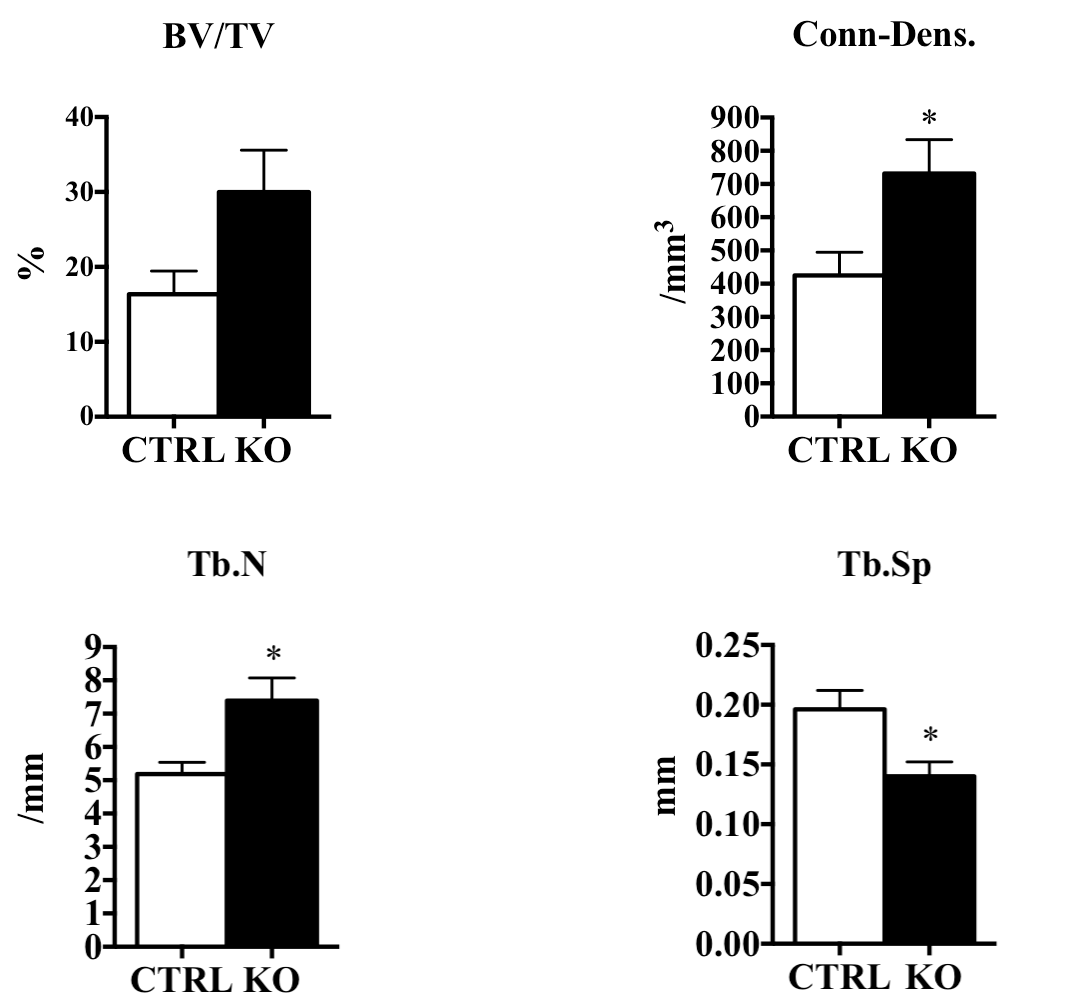

Supplement: S5 Fig — CTRL n = 8; KO n = 8. * = p<0.05. p-values were calculated using an unpaired two-tailed Student’s t-test. (TIF) [file pone.0247199.s005.tif]

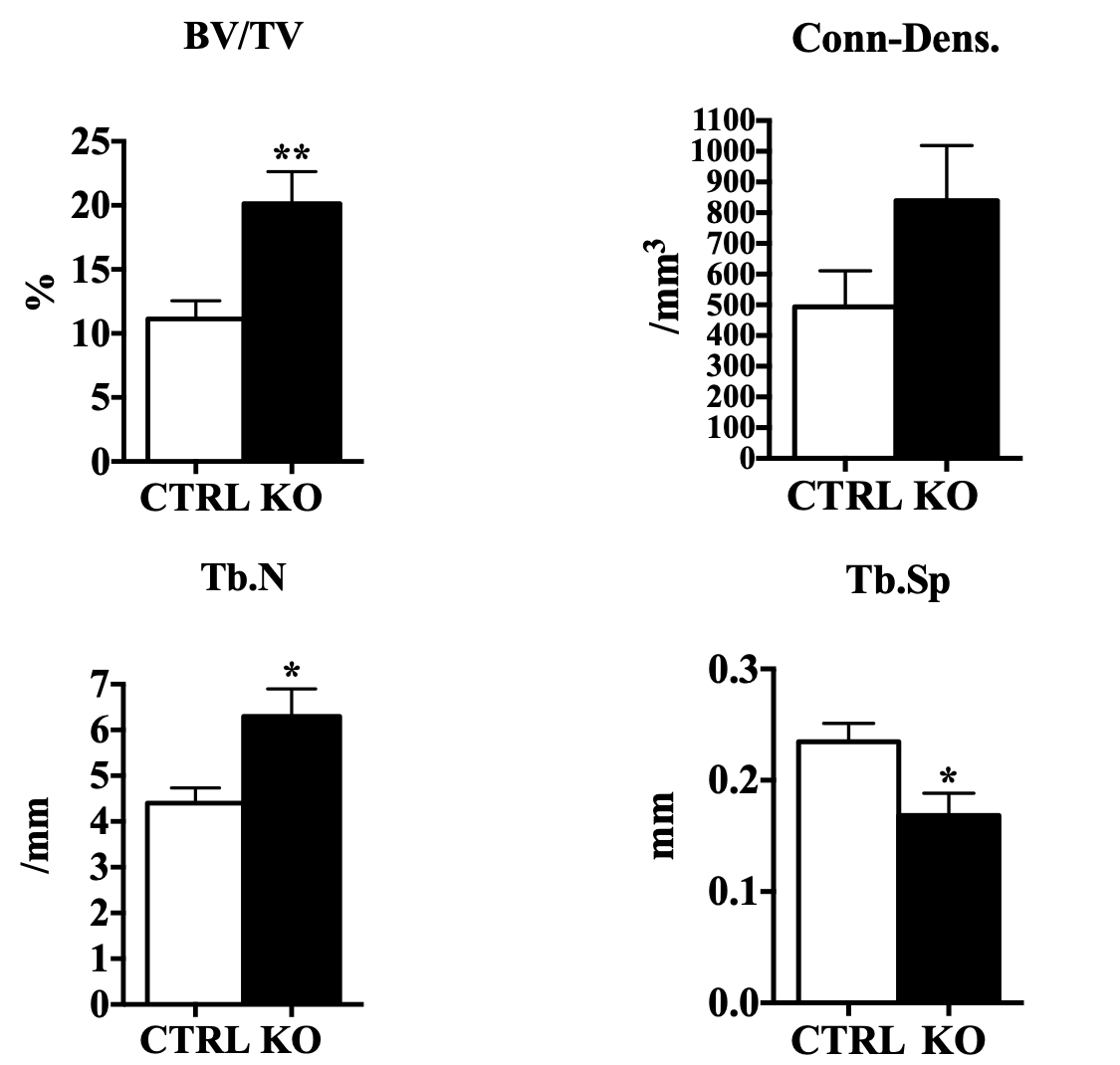

Supplement: S6 Fig — CTRL n = 8; KO n = 8. * = p<0.05. ** = p<0.01. p-values were calculated using an unpaired two-tailed Student’s t-test. (TIF) [file pone.0247199.s006.tif]

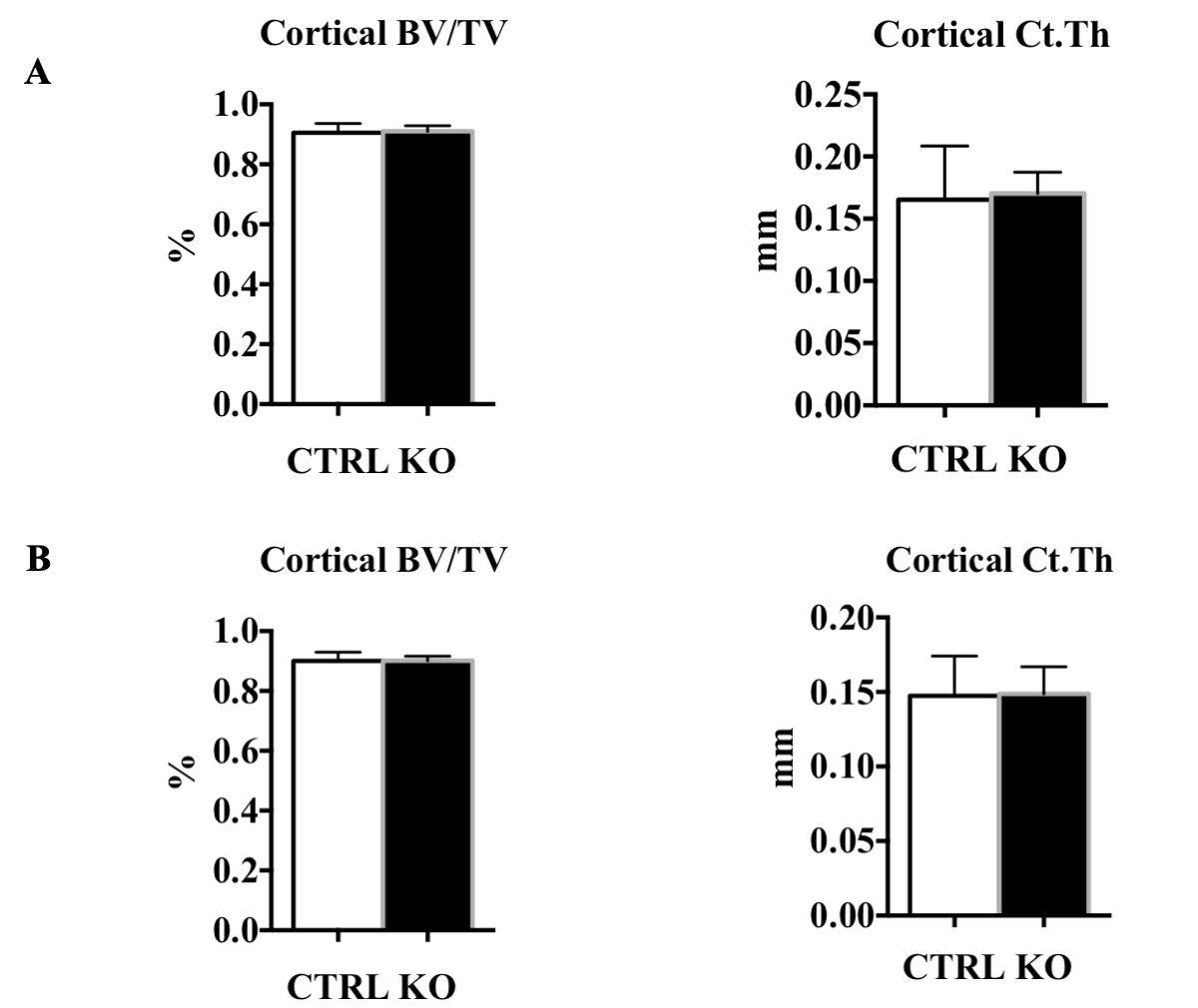

Supplement: S7 Fig — A: MicroCT analysis of femoral cortical bone in 5–6 week old male mice. CTRL n = 8; KO n = 8. B: MicroCT analysis of femoral cortical bone in 5–6 week old female mice. CTRL n = 8; KO n = 8. (TIF) [file pone.0247199.s007.tif]

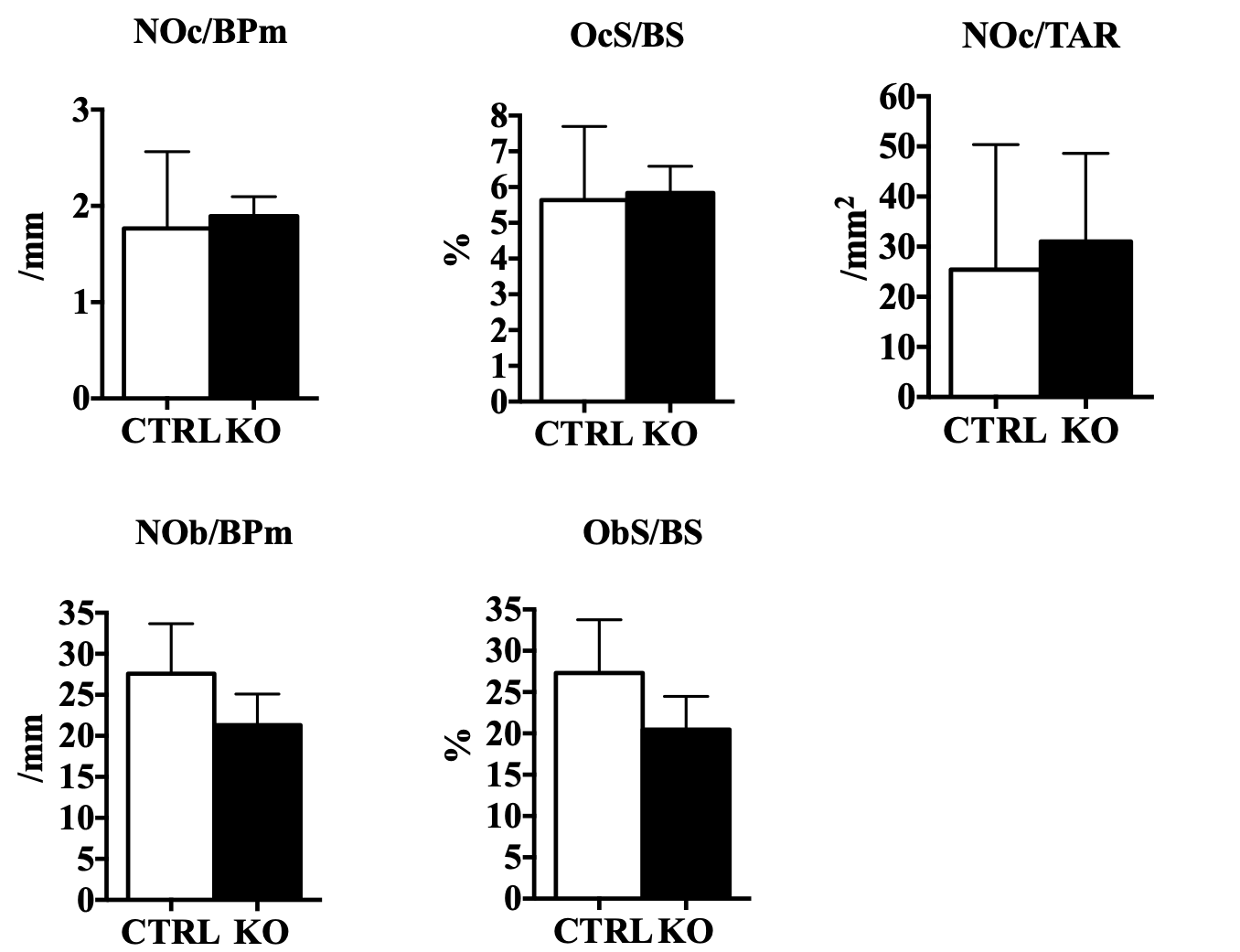

Supplement: S8 Fig — CTRL n = 4; KO n = 3. (TIF) [file pone.0247199.s008.tif]

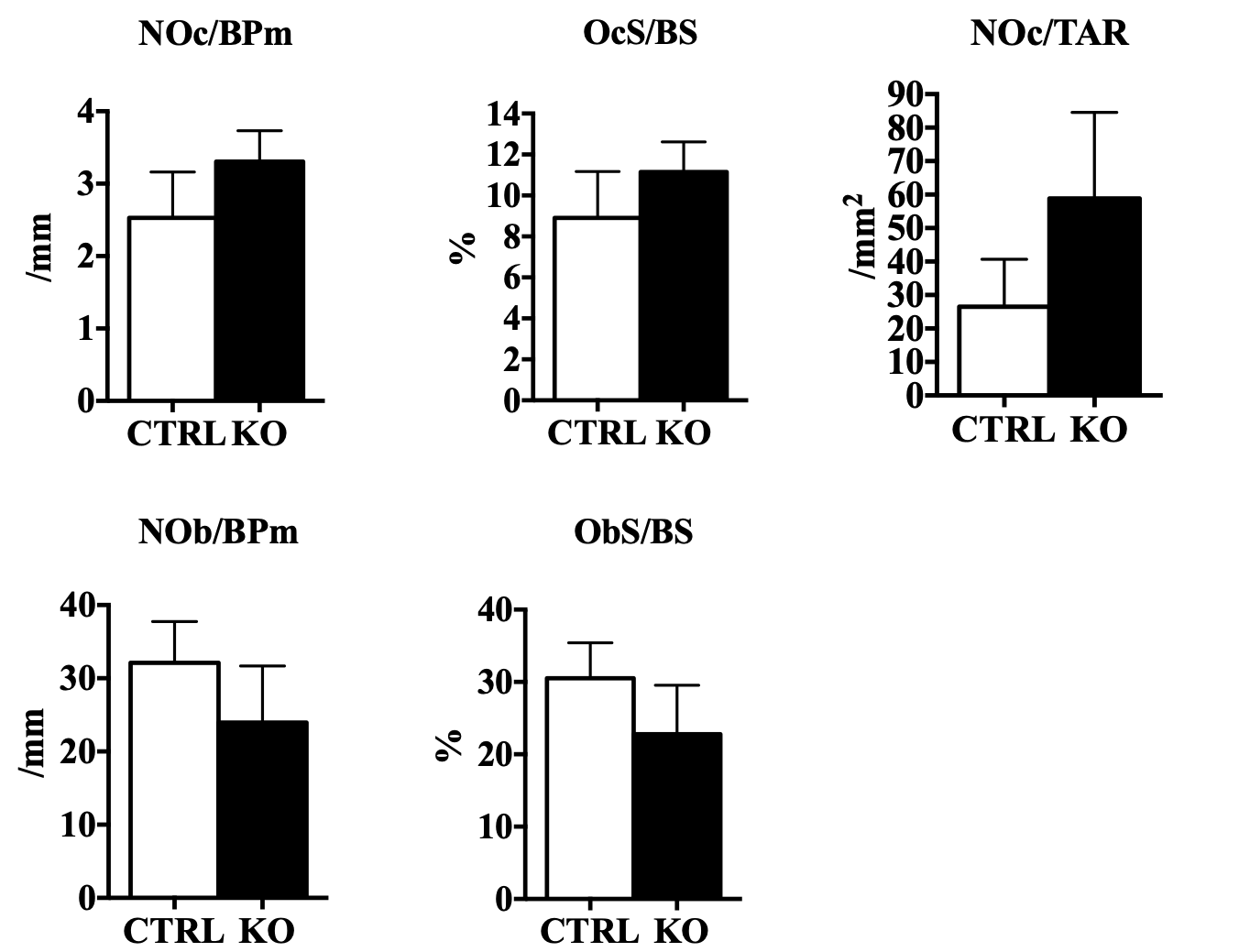

Supplement: S9 Fig — CTRL n = 4; KO n = 4. (TIF) [file pone.0247199.s009.tif]

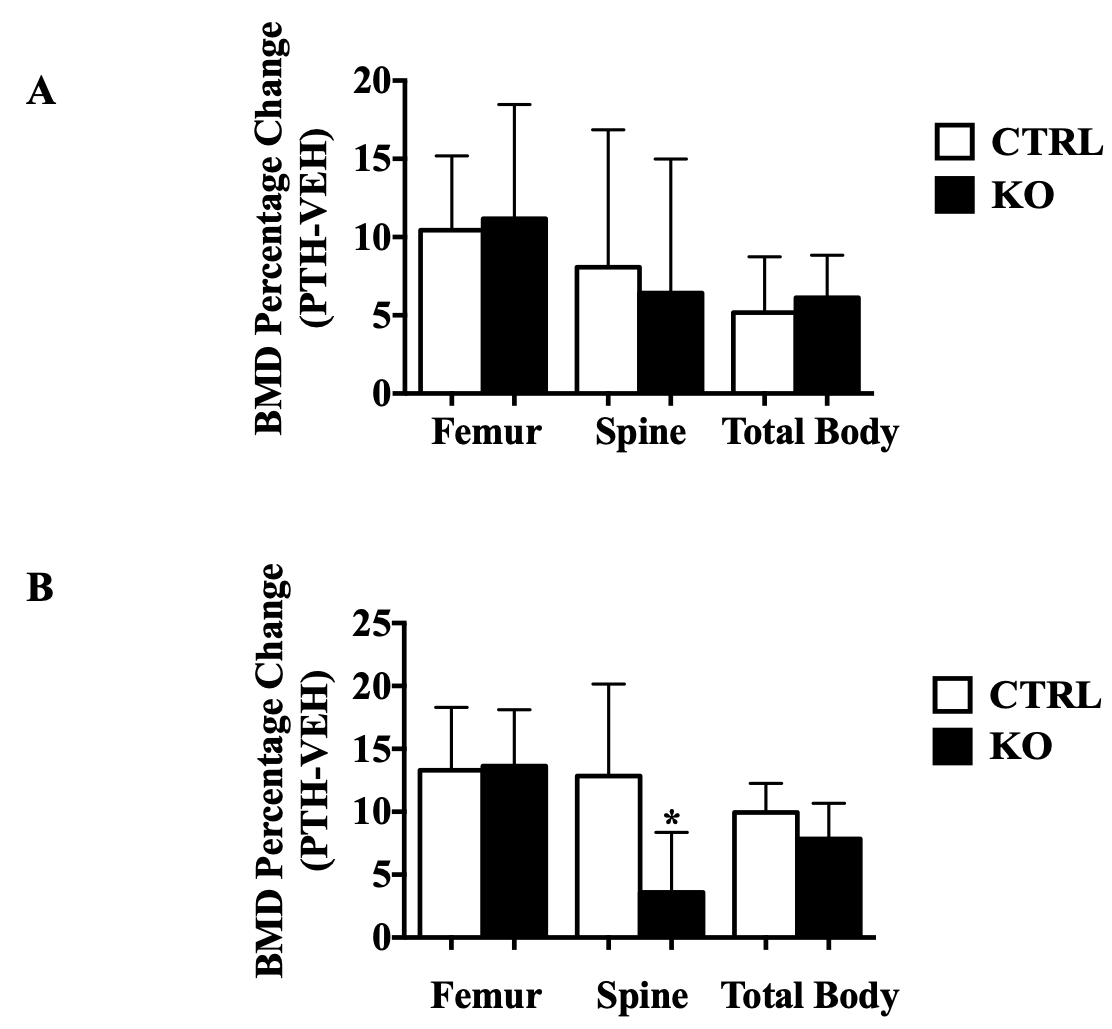

Supplement: S10 Fig — A: male mice; CTRL n = 7; KO n = 6. B: female mice; CTRL n = 7; KO n = 8. * = p<0.05. p-values were calculated using an unpaired two tailed Student’s t-test. (TIF) [file pone.0247199.s010.tif]

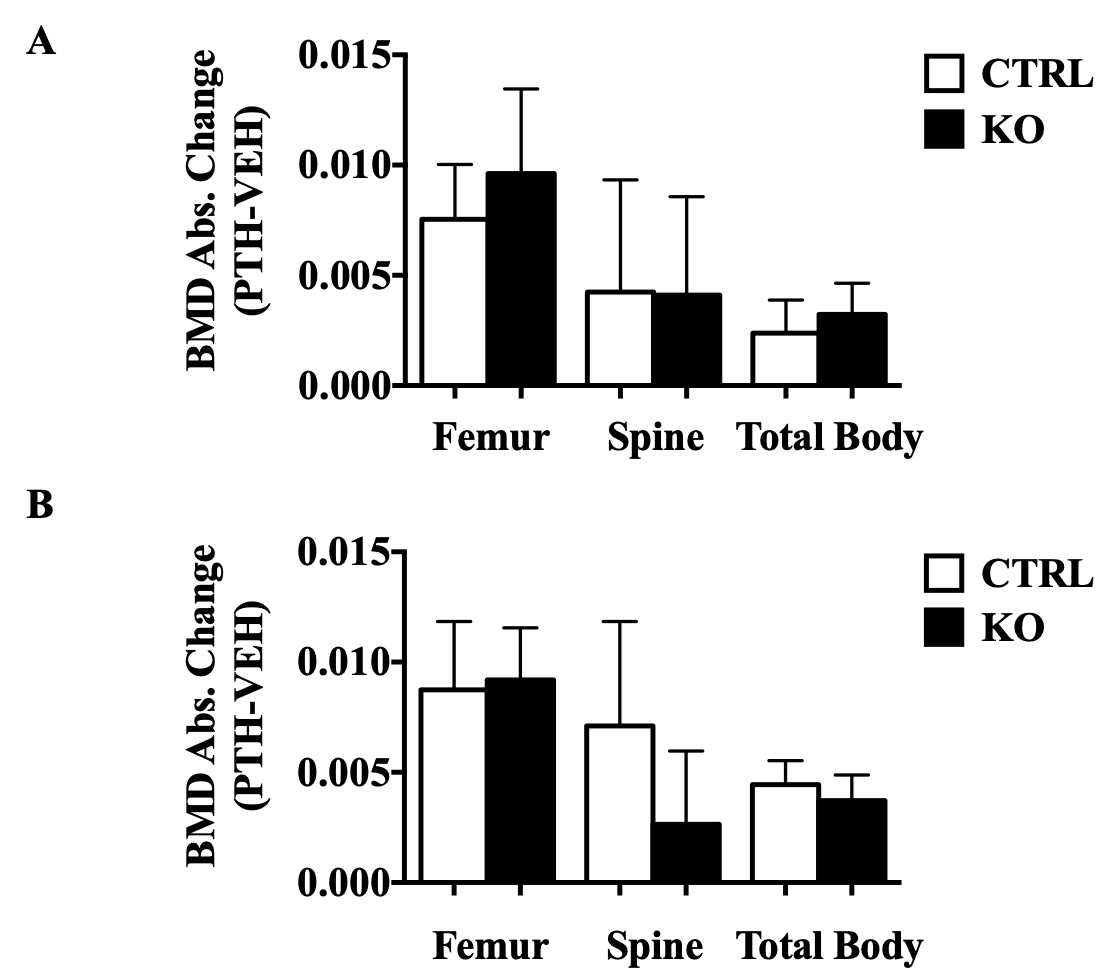

Supplement: S11 Fig — A: male mice; CTRL n = 7; KO n = 6. B: female mice, CTRL n = 7; KO n = 8. (TIF) [file pone.0247199.s011.tif]
