# Supplementary material for: Selective deletion of the receptor for CSF1, c-fms, in osteoclasts results in a high bone mass phenotype, smaller osteoclasts in vivo and an impaired response to an anabolic PTH regimen
Source: PLoS One. 2021 Feb 19;16(2):e0247199. doi: 10.1371/journal.pone.0247199 (PMC7895546; doi:10.1371/journal.pone.0247199)
Supplement: S1 Table — (DOCX) [file pone.0247199.s012.docx]

| **Genotype** | **Hemoglobin**  **(g/dl)** | **WBC**  **(cells/µl)** | **Lymphocytes**  **(cells/µl)** | **Monocytes**  **(cells/µl)** |
| --- | --- | --- | --- | --- |
| **CTRL** | 15±0.1 | 4,260±598 | 3715±544 | 58±8 |
| **KO** | 14.9±0.3 | 3,830±682 | 3274±616 | 55±16 |
| **P value** | 0.8 | 0.6 | 0.6 | 0.8 |

Values are M±SEM. N= 10 for CTRL and N=10 for KO. Five male and five

female animals for each genotype were analyzed
